# Supplementary material for: The mammalian LINC complex component SUN1 regulates muscle regeneration by modulating drosha activity
Source: eLife. 2019 Nov 5;8:e49485. doi: 10.7554/eLife.49485 (PMC6853637; doi:10.7554/eLife.49485)
Supplement: Supplementary file 1. [file elife-49485-supp1.docx]

**Supplementary file 1**: SUN1 ∆7, ∆9 Y2H candidates

and libraries screened

| Candidates | Libraries |
| --- | --- |
| mCIZ1 | uterus/mammary-gland mix |
| DROSHA | uterus/mammary-gland mix |
| DROSHA | total brain |
| mSNCAIP | uterus/mammary-gland mix |
| LOC283767 | testis |
| LmnB2 | macrophage |
| LmnB2 | Embryo |
| LmnB2 | uterus/mammary-gland mix |
| AES | spleen |
| Golga6L1 | testis |
| FBLIM1 | macrophage |
| TRIM17 | spleen |
| TXNDC11 | spleen |
| PSME4 | testis |
| mBICD2 | uterus/mammary-gland mix |
| mCDON | uterus/mammary-gland mix |
| mFHOD1 | Embryo |
| mPHF23 | uterus/mammary-gland mix |
| mPKNOX2 | uterus/mammary-gland mix |
| mVCPIP1 | uterus/mammary-gland mix |
| mNFKB1 | macrophage |
| RINT1 | testis |
| mZBTB1 | macrophage |
| MATR3 | total brain |
| ZFP655 | uterus/mammary-gland mix |
| ZNF238 | total brain |
| CDCA2 | breast cancer |
| SYCE1 | testis |
