# Supplementary material for: The mammalian LINC complex component SUN1 regulates muscle regeneration by modulating drosha activity
Source: eLife. 2019 Nov 5;8:e49485. doi: 10.7554/eLife.49485 (PMC6853637; doi:10.7554/eLife.49485)
Supplement: Supplementary file 2. — LGMD1B: Limb girdle muscular dystrophy type 1B; DCM: Dilated cardiomyopathy; CD: Conduction disease; CD-ARRH: conduction defects with arrhythmias, EDMD; Emery-Dreifuss muscular dystrophy; ICD: implantable cardiac defibrillator, PLD: Partial lipodystrophy. [file elife-49485-supp2.docx]

**Supplementary File 2**. Clinical phenotype and genotypes of *LMNA* mutated patients.

| **Patient Code in Figure 8** | ***LMNA* mutation** | **Phenotype** | **Main clinical features** | | **Age at biopsie**  **(years)** | **Histopathologic features of the muscle biopsy** | **Reference** |
| --- | --- | --- | --- | --- | --- | --- | --- |
|  |  |  | **Onset:**  **symptoms / age (years)** | **Last examination: main features / age (years)** |  |  |  |
| Spo 2 (male) | c.881A>C ;  p.Gln294Pro | EDMD | Frequent falls, toe walking / 5 | Humero-peroneal muscle weakness and wasting, joint contractures (elbows, ankles, spine and neck), cardiomyopathy, PM, heart transplantation planed but died before / 26 | 25 | Biceps brachii; type 1 fibers atrophy, few internalised nuclei, mild endomysial fibrosis, no necrotic or regenerative fibers | ([Bonne et al., 2000](#_ENREF_2); [Brette et al., 2004](#_ENREF_3)) |
| F124-EMD205 (male) | c.936G>C ; p.Gln312His | LGMD1B | Difficulties in running, thin legs / 5 | Proximal muscle weakness in upper (biceps brachii) and lower limbs (psoas), no joint contractures, early (14 years old) and severe cardiomyopathy (heart transplantation at 14 years old) / 18 | 15 | Quadriceps; mild abnormalities (fiber size variation, hypotrophic fibers, endomysial fibrosis, necrotic and regenerative fibers) | ([Ben Yaou et al., 2005](#_ENREF_1); [Brette et al., 2004](#_ENREF_3)) |
| EDM674 (male) | c.1129C>T ;  p.Arg377Cys | EDMD | Inability to run, dyspnea / childhood | Humero-peroneal muscle weakness, mild Achilles tendons contractures, cardiomyopathy, PM then ICD, heart transplantation planed but died before / 43 | 42 | Quadriceps; fiber size variation, hypotrophic fibers, type 2 fiber predominance, no internalised nuclei, no necrotic or regenerative fibers | ([Deconinck et al., 2010](#_ENREF_4)) |
| LMNA-3  (female) | c.178C>G ; p.R60G | DCM-CD + PLD | Increased fat in face & neck, limbs lipoatrophy  / adolescence | Cardiomyopathy with CD-ARRH, ICD + limbs lipoatrophy, face fat sparing + dyslipidemia and diabetes mellitus / 44 | 43 | Variable fibre size,  few internalized nuclei | ([van der Kooi et al., 2002](#_ENREF_5)) |
| LMNA-4  (female) | c.1129C>T ; p.R377C | LGMD1B | Difficulties in climbing stairs / 42 | Pelvic and scapular muscle weakness, axial muscle wasting, no contractures, cardiomyopathy with CD, ICD / 56 | 52 | Variable fibre size,  few internalized nuclei  few ragged red fibers  predominance of type I fibres | ([Ben Yaou et al., 2005](#_ENREF_1)) |
| EMD706 (female) | c.810+1G>A (c.IVS4+1G>A) | EDMD | Unknown symptoms of onset / adolescence | Diffuse muscle weakness and wasting with joint contractures (elbows, hips, spine, neck), cardiomyopathy, PM, heart transplanted / 32 | 36 | Deltoid; hypotrophic fibers, regenerative fibers | (Van den Berg P, Richard P, Bonne G, personnal communication) |

LGMD1B: Limb girdle muscular dystrophy type 1B; DCM: Dilated cardiomyopathy; CD: Conduction disease; CD-ARRH: conduction defects with arrhythmias, EDMD; Emery-Dreifuss muscular dystrophy; ICD: implantable cardiac defibrillator, PLD: Partial lipodystrophy.

Ben Yaou, R., Becane, H.M., Demay, L., Laforet, P., Hannequin, D., Bohu, P.A., Drouin-Garraud, V., Ferrer, X., Mussini, J.M., Ollagnon, E.*, et al.* (2005). [Autosomal dominant limb-girdle muscular dystrophy associated with conduction defects (LGMD1B): a description of 8 new families with the LMNA gene mutations]. Rev Neurol (Paris) *161*, 42-54.

Bonne, G., Mercuri, E., Muchir, A., Urtizberea, A., Becane, H.M., Recan, D., Merlini, L., Wehnert, M., Boor, R., Reuner, U.*, et al.* (2000). Clinical and molecular genetic spectrum of autosomal dominant Emery-Dreifuss muscular dystrophy due to mutations of the lamin A/C gene. Ann Neurol *48*, 170-180.

Brette, S., Penisson-Besnier, I., Dupuis, J.M., Bonne, G., and Victor, J. (2004). [Cardiac manifestations of laminopathies]. Arch Mal Coeur Vaiss *97*, 973-977.

Deconinck, N., Dion, E., Ben Yaou, R., Ferreiro, A., Eymard, B., Brinas, L., Payan, C., Voit, T., Guicheney, P., Richard, P.*, et al.* (2010). Differentiating Emery-Dreifuss muscular dystrophy and collagen VI-related myopathies using a specific CT scanner pattern. Neuromuscul Disord *20*, 517-523.

van der Kooi, A.J., Bonne, G., Eymard, B., Duboc, D., Talim, B., Van der Valk, M., Reiss, P., Richard, P., Demay, L., Merlini, L.*, et al.* (2002). Lamin A/C mutations with lipodystrophy, cardiac abnormalities, and muscular dystrophy. Neurology *59*, 620-623.
