# Supplementary material for: The mammalian LINC complex component SUN1 regulates muscle regeneration by modulating drosha activity
Source: eLife. 2019 Nov 5;8:e49485. doi: 10.7554/eLife.49485 (PMC6853637; doi:10.7554/eLife.49485)
Supplement: Supplementary file 3. [file elife-49485-supp3.docx]

| Primer name (usage) | Forward | Reverse |
| --- | --- | --- |
| Sun1 (PCR nucleoplasmic domain) | ATGGACTTTTCTCGGCTGCACACG | CCCGGCAGCTCTAGTCCTTCGCAG |
| Rtl1as (RT with Accuscript) |  | ACTACACGCAGATGGTACCAGAGC |
| Rtl1as (qPCR with Roche universal probe #16) | TCTTGCCAGTTTCGTCGTC | CAGACATCACTGGGTCATTCC |
| Rtl1 (qPCR) | GGAGCTAACGTGACCAAGTCT | CCAAAACCACTGTTAGCGCC |
| Aberrant pre-miR127 (qPCR) | ATGGACTGAAGGAGTAGAAACTG | AGACTTCCGACCAGCCAAGC |
| Mhy3 (qPCR) | CTTCACCTCTAGCCGGATGGT | AATTGTCAGGAGCCACGAAAAT |
| Dlk1 (qPCR) | GACGGGAAATTCTGCGAAATAGAC | GTCCACGCAAGTTCCATTGTTG |
| Meg3 (qPCR) | CGAGGACTTCACGCACAACAC | CCACGCAGGATTCCAGATGATG |
| Pax7 (qPCR) | GCGAGAAGAAAGCCAAACAC | GTCGGGTTCTGATTCCACAT |
| Drosha (qPCR) | CTGAGGACAGAAGGGAAAGAG | GTCCTTTCCCACAGCCTATCC |
| miR-127 (RLM-RACE) | GeneRacer™ 5′ Nested Primer | CCCATGCCCCTGAAGTCGACTGGA |
| miR-433 (RLM-RACE) | GeneRacer™ 5′ Nested Primer | ACTACACGCAGATGGTACCAGAGC |
| miR-431 (RLM-RACE) | GeneRacer™ 5′ Nested Primer | CCCAGCTCGAGAGCTGGCTGAGCT |

**Supplementary File 3**: Primer sequences used in experiments
