# Supplementary material for: The mammalian LINC complex component SUN1 regulates muscle regeneration by modulating drosha activity
Source: eLife. 2019 Nov 5;8:e49485. doi: 10.7554/eLife.49485 (PMC6853637; doi:10.7554/eLife.49485)
Supplement: Transparent reporting form [file elife-49485-transrepform.docx]

***eLife’s* transparent reporting form**

We encourage authors to provide detailed information *within their submission* to facilitate the interpretation and replication of experiments. Authors can upload supporting documentation to indicate the use of appropriate reporting guidelines for health-related research (see [EQUATOR Network](http://www.equator-network.org/%20)), life science research (see the [BioSharing Information Resource](https://biosharing.org/)), or the [ARRIVE guidelines](http://www.plosbiology.org/article/info:doi/10.1371/journal.pbio.1000412) for reporting work involving animal research. Where applicable, authors should refer to any relevant reporting standards documents in this form.

If you have any questions, please consult our Journal Policies and/or contact us: [editorial@elifesciences.org](mailto:editorial@elifesciences.org).

**Sample-size estimation**

- You should state whether an appropriate sample size was computed when the study was being designed
- You should state the statistical method of sample size computation and any required assumptions
- If no explicit power analysis was used, you should describe how you decided what sample (replicate) size (number) to use

Please outline where this information can be found within the submission (e.g., sections or figure legends), or explain why this information doesn’t apply to your submission:

We determined biological sample size is 3 mice for each genotype for non-surgical experiments. For the muscle grafting experiments (surgery) there were a minimum of 3 or more for each genotype (all males) depending on the numbers of male mice in each litter.

Figure 1:

The biological sample size for Sun1 isoforms in the different mouse tissues was (n=1), and for determining the Sun1 isoform distribution in muscle and heart (n=3).

Figure 2:

The biological sample size (n) is indicated in Figure legend 2; with the WT mice number (n=7) and Sun1-/- (ko) number was (n=5). Reciprocal muscle grafts of WT/ko mice (n=6) and ko/WT mice (n=7). The statistical test used was mean +/-SEM and p value. Myofiber size (CSA) quantification was performed in all grafts and plotted as size distribution curves. Immunofluorescence studies was performed for all samples to tally gene expression and genotyped samples.

Figure 3:

The Immunofluorescence studies were performed on WT derived myoblasts and Sun1-/- (ko) derived myoblasts from biological samples (n=3 each).

The immunoprecipitation experiments were each performed at least 3 times.

Figure 4:

MicroRNA microarray and qPCR studies was performed on WT derived myoblasts and Sun1-/- (ko) derived myoblasts from 3 different biological samples (n=3 each). Technical triplicates were performed for all qPCR experiments.

Figure 5:

RLM-RACE, qPCR was performed on 3 samples each from WT and Sun1 -/- (ko) tissues. In vitro Drosha processing assay was performed 3 times.

Figure 6:

The biological sample sizes are indicated in figures, wit htechnical triplicates for all qPCR experiments.

Figure 7:

The biological sample sizes are indicated in figures. Immunofluorescence studies were performed for all samples to tally gene expression and genotyped samples.

Supp Figure 2:

Biological sample size is indicated in figures.

Supp Figure 3:

The Immunofluorescence studies were performed on WT derived myoblasts and Sun1 -/- (ko) derived myoblasts from biological samples (n=3 each).

Supp Figure 4:

Human biological sample numbers and origin is documented in sup. Table 2.

**Replicates**

- You should report how often each experiment was performed
- You should include a definition of biological versus technical replication
- The data obtained should be provided and sufficient information should be provided to indicate the number of independent biological and/or technical replicates
- If you encountered any outliers, you should describe how these were handled
- Criteria for exclusion/inclusion of data should be clearly stated
- High-throughput sequence data should be uploaded before submission, with a private link for reviewers provided (these are available from both GEO and ArrayExpress)

Please outline where this information can be found within the submission (e.g., sections or figure legends), or explain why this information doesn’t apply to your submission:

Muscle grafting experiments were performed at least 3 times to obtain sufficient biological replicate numbers for histology/immunofluorescence and qPCR analyses.

Outliers are included in the dot plots for the muscle grafting experiments.

We did not encounter outliers in the non-surgical samples.

**Statistical reporting**

- Statistical analysis methods should be described and justified
- Raw data should be presented in figures whenever informative to do so (typically when N per group is less than 10)
- For each experiment, you should identify the statistical tests used, exact values of N, definitions of center, methods of multiple test correction, and dispersion and precision measures (e.g., mean, median, SD, SEM, confidence intervals; and, for the major substantive results, a measure of effect size (e.g., Pearson's r, Cohen's d)
- Report exact p-values wherever possible alongside the summary statistics and 95% confidence intervals. These should be reported for all key questions and not only when the p-value is less than 0.05.

Please outline where this information can be found within the submission (e.g., sections or figure legends), or explain why this information doesn’t apply to your submission:

We used mean +/- SEM and p<0.05 as being significantly different.

(For large datasets, or papers with a very large number of statistical tests, you may upload a single table file with tests, Ns, etc., with reference to sections in the manuscript.)

**Group allocation**

- Indicate how samples were allocated into experimental groups (in the case of clinical studies, please specify allocation to treatment method); if randomization was used, please also state if restricted randomization was applied
- Indicate if masking was used during group allocation, data collection and/or data analysis

Please outline where this information can be found within the submission (e.g., sections or figure legends), or explain why this information doesn’t apply to your submission:

NA

**Additional data files (“source data”)**

- We encourage you to upload relevant additional data files, such as numerical data that are represented as a graph in a figure, or as a summary table
- Where provided, these should be in the most useful format, and they can be uploaded as “Source data” files linked to a main figure or table
- Include model definition files including the full list of parameters used
- Include code used for data analysis (e.g., R, MatLab)
- Avoid stating that data files are “available upon request”

Please indicate the figures or tables for which source data files have been provided:

None
